# The first description of a singular case of synchronous chronic myelomonocytic leukemia and diffuse large b‐cell lymphoma

**DOI:** 10.1002/ccr3.3817

**Published:** 2021-09-21

**Authors:** Alessandra Romano, Michele Giusti, MaryAnn Di Giorgio, Giovanni Lumera, Nunziatina Laura Parrinello, Sebastiano Cosentino, Massimo Ippolito, Loredana Villari, Giuseppe Alberto Palumbo, Francesco Di Raimondo, Salvatore Santo Signorelli

**Affiliations:** ^1^ Dipartimento di Chirurgia e Specialità Medico Chirurgiche Sezione di Ematologia Università degli Studi di Catania Catania Italy; ^2^ Department of Clinical and Experimental Medicine Università degli Studi di Catania Catania Italy; ^3^ UO Medicina Generale AOU Policlinico di Catania, Presidio Rodolico Catania Italy; ^4^ UOC Ematologia AOU Policlinico di Catania, Presidio Rodolico Catania Italy; ^5^ Dipartimento Tecnologie Avanzate UOC Medicina Nucleare ‐ Centro PET AOE, “Cannizzaro” Catania Catania Italy; ^6^ UO Anatomia Patologica AOU Policlinico di Catania, Presidio San Marco Catania Italy; ^7^ Dipartimento di Scienze Mediche, Chirurgiche e Tecnologie Avanzate “G.F. Ingrassia” Sezione di Ematologia Università degli Studi di Catania Catania Italy

**Keywords:** CMML, filgrastim, lymphoma, monocytes, R‐CHOP, supportive care

## Abstract

In CMML, neoplastic monocytes can be distinguished based on their immunophenotype. Supportive care myeloid growth factors in concomitant extranodal non‐Hodgkin Lymphoma are safe.

## Graphical abstract

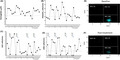

## 1 INTRODUCTION

Chronic myelomonocitic leukemia (CMML) is a myeloid clonal hematopoietic malignancy, characterized by a persistent increase of absolute monocyte count (AMC>=1 + 10^9/L), accounting for more than 10% of the white blood cells, circulating in peripheral blood.^1^

According to WHO 2016 classification, CMML is diagnosed when monocytosis persists for at least 3 months and other causes of monocytosis have been excluded; alternatively, a flow cytometry assay excluding reactive monocytosis with at least 94% of classical CD14^++^CD16^‐^ monocytes circulating in peripheral blood can be conclusive for the diagnosis.^2^, ^3^, ^4^

CMML is a rare disease, affecting preferentially elderly male patients, but associated with a more favorable course in young patients.^1^, ^5^

As recently described, CMML raises from the combination of hypersensitivity of myeloid precursors to granulocyte‐macrophage colony‐stimulating factor, myeloid cells dysplasia, and ineffective hematopoiesis.^3^, ^6^, ^7^ Recent advances in molecular profiling lighted the multistep dynamics of CMML pathogenesis, which can be anticipated by oligomonocytic CMML, in which AMC does not reach a diagnostic level but all other criteria for CMML are fulfilled, or other clonal and nonclonal conditions, including idiopathic monocytosis (IMUS) and clonal monocytosis of unknown significance (CMUS). In some patients, CMML is anticipated by a diagnosis of clonal cytopenia of unknown significance (CCUS), clonal hematopoiesis of indeterminate potential (CHIP), and idiopathic cytopenia of undetermined significance (ICUS).^5^

To make the picture more complex, in a few patients, CMML is associated with other hematological disease, including lymphoproliferative diseases such as monoclonal gammopathy if uncertain significance (MGUS), monoclonal B‐cell lymphocytosis (MBL), multiple myeloma (MM), or non‐Hodgkin T‐ and B‐cell lymphomas.^1^ In most cases, the diagnosis of lymphoid neoplasm is detected first and CMML is considered a post‐treatment disease, but there is lack of knowledge due to the anecdotal nature of these observations.

Herein, we report the first case described in literature about the synchronous diagnosis of large B‐cell lymphoma (DLBCL) and CMML.

### 1.1 Case presentation

A 68‐year‐old Caucasian man was referred by the emergency room for anemia, secondary to blood loss due to several episodes of hematemesis and melena. He had a history of not compensated mellitus diabetes type II, diverticulitis, essential hypertension and benign prostatic hyperplasia (BPH) and complained of chronic inflammatory pain for ureteral stenosis, weight loss (3 kg in the last month) and fever, without nausea or abdominal pain.

On physical examination, his vital signs were normal, and no palpable lympho‐adenopathy or splenomegaly. The review of his medical records from the previous two years showed a progressive increase in leucocytosis, including monocytosis, which reached a peak of 5,040 absolute monocyte cells (AMC)/mmc, in absence of blast at the evaluation of the peripheral blood smear.

Blood count examination showed severe anemia (Hb 6.4 g/dL) that required repeated blood transfusions, thrombocytopenia (platelet count at baseline 64,000/mmc), moderate leucocytosis (white blood cells, WBC, 13,900/mmc), with absolute monocytosis (AMC 3,300/mmc). Laboratory investigations showed elevated levels of lactate dehydrogenase, C‐reactive protein, and beta2 microglobulin. None of cancer markers neither acute infections tests were found as positive.

#### 1.1.1 Second level examinations: methods and results

The esophagus‐gastroscopic examination (EGE)^8^, ^9^ demonstrated a vegetative, ulcerated mass in the stomach antrum and body, worsened a month after with progressive extension and thickening of ulcerated areas.

**FIGURE 1:**
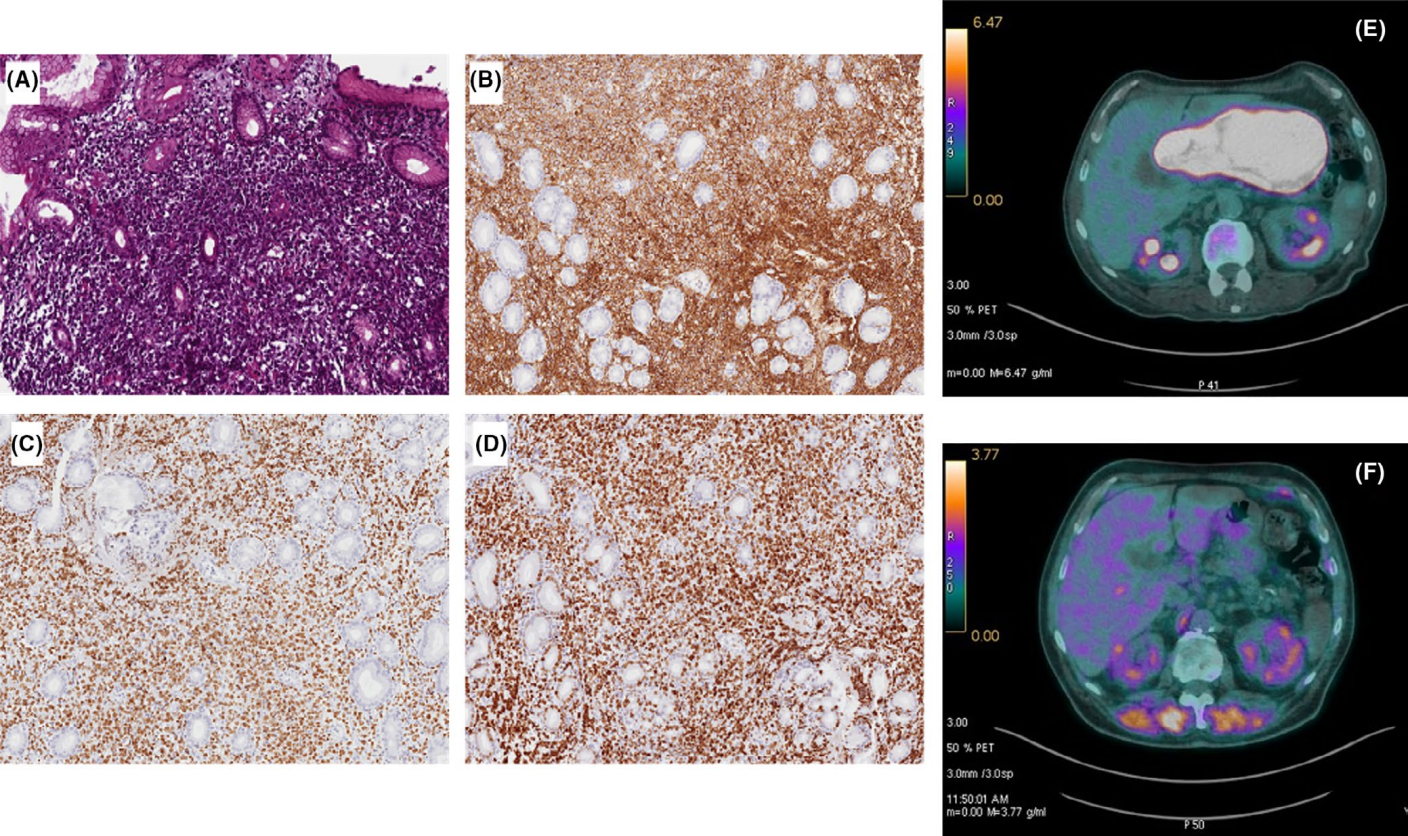
Immunohistochemical and imaging features of DLBLC Photomicrographs of the stomach histology and immunohistochemistry supporting the diagnosis of diffuse large B‐cell lymphoma (DLBCL). **(A)** Hematoxylin and eosin showed a diffuse infiltration of the lamina propria, with replacement of most of the glands by large‐sized lymphoid cells with vesicular nuclei replacing the normal gastric structure. Immunohistochemistry showed a positive immunostaining (brown) of the lymphocytes with the B‐cell antibody CD20 **(B)**, Bcl‐6 **(C)**, and high ki67 index **(D)**. Staging procedures included CT‐PET **(E)** scan, repeated after first four months of treatment **(F)**, compatible with a partial remission.

Histological examination (HE) of the gastric biopsy showed diffuse infiltration of the lamina propria, with replacement in most glands by large lymphoid cells with vesicular nuclei (Figure 1A). The immune histochemical profile was positive for LCA, CD20, PAX5, Bcl‐6, and Bcl2 neoplastic cells (Figure 1B, C), associated with high proliferation rate (90% ki67 index), and negative for CD3, CD5, CD10, Cyclin D1, and CD23 (Figure 1 A‐D), in absence of translocations involving MYC, BCL‐2, and BCL‐6,^10^ thus conclusive for the diagnosis of diffuse large B‐cell lymphoma (DLBCL).

Computed tomography (CT) imaging of the chest and abdomen showed a thickening of stomach antrum and body, associated with enlarged lymph nodes (max diameter 18 mm) in the perigastric adipose tissue, and two suspect lesions in the left adrenal gland, due to increased enhancement after injection of contrast dye in absence of hepatosplenomegaly (Figure 1E).

**FIGURE 2:**
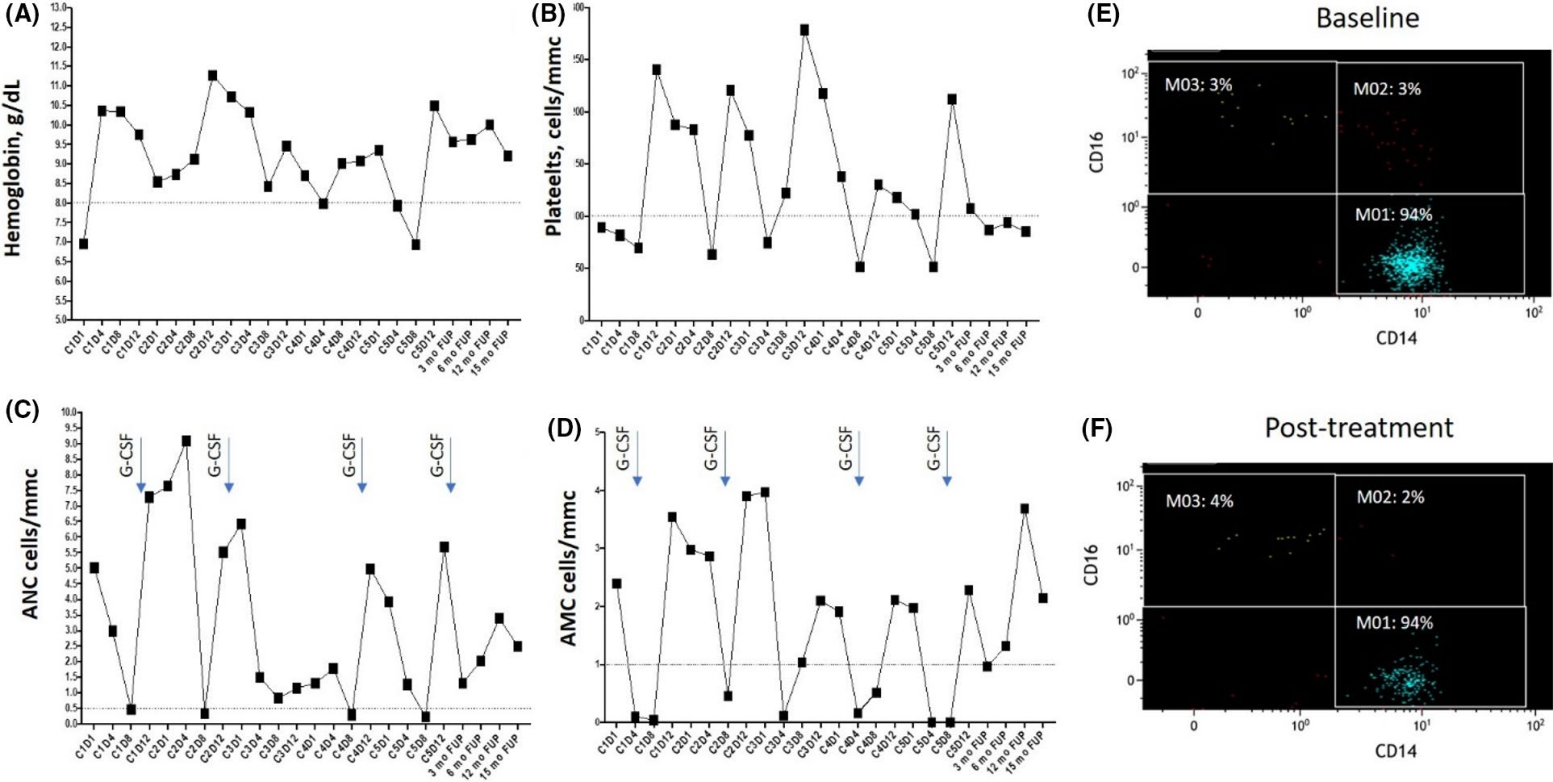
Blood counts in peripheral blood during before, during, and after treatment for DLBCL Hematological parameters in peripheral blood during before, during, and after treatment for DLBCL are reported, referred to hemoglobin, measured in g/dL **(A)**, platelet count/mmc **(B)**, absolute neutrophil count **(C),** and absolute monocyte count **(D)**. Arrows in **(C)** and **(D)** indicate the start of supportive care with G‐CSF. Abbreviations: C, cycle, D, day. Monocyte subset repartition based on multiparameter flow cytometry analysis of monocyte (MO) subsets at baseline **(E)** and after treatment specific for DLBCL **(F)**, showing an increased fraction of classical monocytes (>94%).

18‐F‐fluoro‐2‐deoxyglucose (FDG) positron emission imaging showed increased uptake at the level of stomach antrum (standardized uptake value, SUVmax = 34) and skeleton as by bone marrow activation, without involvement of left adrenal gland. The lymphoma staging was completed by the bone marrow examination, which excluded the presence of lymphoproliferative infiltrate, showing dysgranulopoiesis, associated with an increase in the immature granulocyte and monocyte compartment (MPO+, CD33+, CD14+, CD68+) and fibrosis grade 1 (Figure 2A), conclusive for chronic myelomonocytic leukemia (CMML).

As CMML is invariably associated with monocytosis, all causes of reactive monocytosis should be investigated before of a conclusive diagnosis.^4^ Based on the first report of Dr Seligmoglu‐Buet and colleagues,^3^ the presence of > 94% of CD14^+^CD16^−^ classical monocytes (MO1s) in the peripheral blood is a strong and better predictor of CMML than the 1 × 10^9^/L cutoff currently used by the WHO. Also in our case, the immune phenotype of monocytes circulating in peripheral blood was helpful, showing increased absolute numbers of CD14^+^CD16^−^ classical MO1s and inflammatory CD14^+^CD16^+^ MO2s, associated with lack of CD14^low^CD16^+^MO3s (Figure 2 E), confirmed at the bone marrow level.

Conventional cytogenetics showed a normal XY karyotype, and molecular profiling identified the single mutation in ASXL1 gene (exon 12), generally associated with inferior outcome in de novo CMML.^11^ Thus, the patient has a high‐risk DLBCL, according to the International Prognostic Index, associated with a synchronous diagnosis of CMML‐0,^1^ Intermediate‐1 risk according to CPSS‐mol score.^11^

The management and treatment of lymphoid neoplasms presenting with concomitant CMML is challenging, due to the lack of large series, and at the best of our knowledge, no other cases of DLBCL have been described before. Due to the aggressive clinical course of DLBCL, we planned six cycles of R‐CHOP‐21 treatment consisting of intra venous injection on day 1 (D1) of Rituximab 375 mg/mq (given 1400 mg subcutaneously from the second cycle onwards), cyclophosphamide 750 mg/mq, vincristine 1.4 mg/mq, and prednisone 100 mg per os on days 1 to 5. Prednisone was omitted in the first three cycles due to uncontrolled diabetes and risk of perforation, and dosage halfed at cycles 4 and 5. Due to severe constipation, vincristine was given at 1 mg flat dose at cycles 1‐2‐3 and omitted at cycles 4‐5. The last cycle was not given due to dysphagia, abdominal pain, and acute kidney failure due to ureteral stenosis.

Supportive care consisted of prophylaxis with cotrimoxazole 800 mg bid twice a week, acyclovir 800 mg bid, 40,000 UI erythropoietin zeta given once a week, and 30MU filgrastim given at days + 8+9 of each cycle, and it was sufficient to avoid delays in recycling. Hematological toxicities included neutropenia grade 3, while anemia and thrombocytopenia improved during the course of treatment (Figure 2). No hematological toxicities were expected and included severe stipsis due to vincristine exposure, dysphagia, and asymptomatic increased of transaminases due to doxorubicine. No active infections were registered.

At the end of the treatment, the gastric lesion was reduced more than 50%, as documented by CT‐PET scans (Figure 1F). After 6 months from the end of treatment, a weak uptake of 18‐DG (SUV max 5.3) was still found, but it could be due to lack of resolution of inflammatory status. Bone marrow biopsy showed no residual CMML, and the mutation of ASXL1 was no longer found, despite the persistence of monocytosis, due to increased absolute and percentage numbers of CD14 + CD16− classical MO1s (Figure 2 F), and anemia.

At 15 months follow‐up, the patient is alive, without gastrointestinal discomfort, maintaining CMML‐0 diagnosis, not requiring any additional supportive care.

## 2 DISCUSSION

The synchronous diagnosis of CMML and lymphomas is a quite rare event. Anecdotal cases of association with angioimmunoblastic T‐cell lymphoma, plasma cell disorders, and marginal B‐cell cell lymphoma have been described.^12^ This is the first clinical description of association of CMML with DLBCL.

About 40% of CMML patients carry mutations in the chromatin remodeling gene ASXL1,^13^ which confers inferior clinical outcome.^1^ In our patient, molecular profiling disclosed a mutation in the ASXL1. Since some mutations, such as those involving ASXL1 and DNMT3a genes, could be associated with clonal hematopoiesis of indeterminate potential and increase the risk of a second blood malignancy,^14^, ^15^ we looked for their involvement in the gastric biopsy. The lack of overlapping mutation profile status between CMML and DLBCL suggested that these two hematological malignancies were not related one each other. After specific chemotherapy for DLBCL, ASXL1 mutation was not detectable anymore in the CMML cells, despite persistence of monocytosis, suggesting that the treatment eradicated a probable founder subclone.^16^, ^17^ The immune phenotype^4^ and the persistence after treatment excluded the expansion of monocytic myeloid derived suppressor cells.^18^

In nontransplantable patients, both diseases require separate treatment plans.^1^ The choose of treatment reflected the need to treat first of all those symptoms related to gastric localized lymphoma. Despite a suspicious lesion in the adrenal gland, we did not perform any prophylaxis against secondary central nervous system disease, a complication that occurs in approximately 5% of patients with DLBCL, generally within the first year after the diagnosis, due to low platelet count and the increased risk of hematological toxicity. We paid more attention on the administration of GCSF, to avoid the stimulation of the growth of myeloid precursors. In lack of specific guidelines for the supportive care measures in CMML patients, we took advantage of more mature experience in managing myelodysplastic syndromes (MDS) administrating erythropoietin analogs for the treatment of anemia and prophylactic antibiotics and antivirals.^5^ Based on National Cancer Center Network (NCCN) treatment guidelines, the routine use of G‐CSF should be avoided in patients with MDS, but at the same time G‐CSF injections are recommended as prophylaxis in case of neutropenia with recurring, serious infections, or during infectious episodes. We considered to limit the risk of any infectious disease using an on‐demand policy for filgrastim injection, based on the ANC count evaluated at different time points during the cycle. In this way, hematic crasis improved overtime while no infections occurred.

## 3 CONCLUSIONS

In conclusion, CMML is a rare myeloid disorder that is rarely associated with NHL, as reported in this uncommon presentation. The immune phenotype and the persistence after treatment excluded the expansion of monocytic myeloid derived suppressor cells. It is probable that age relates with the increasing of genetic mutations that can involve also lymphoid maturation lines. A personalized approach is required to personalize the treatment to reduce toxicity and increase the probability of long‐term remission, including on‐demand filgrastim injection.

## 4 ETHICS STATEMENT

This study was conducted in respect of Declaration of Helsinki.

## CONFLICT OF INTEREST

AR and FDR received honoraria from Amgen, Novartis, and Takeda. NLP and GAP received honoraria from Novartis. All the other authors do not have any conflicts of interest.

## AUTHOR CONTRIBUTION

AR and MG.: described the case and performed literature research. MDG and GL: performed bone marrow aspirate and prepared figures. NLP: performed flow cytometry assays. SC and MI: performed radiological scans. LV: performed immunohistochemistry. GAP: coordinated supportive care. FDR, SS, and AR: designed the study and wrote the manuscript.

## Data Availability

No further data are available.

## References

[1] Valent P, Orazi A, Savona MR, et al. Proposed diagnostic criteria for classical chronic myelomonocytic leukemia (CMML), CMML variants and pre‐CMML conditions. Haematologica. 2019;104(10):1935‐1949. 31048353 10.3324/haematol.2019.222059 PMC6886439

[2] Vazquez R, Roussel M, Badaoui B, et al. High sensitivity of the Hematoflow™ solution for chronic myelomonocytic leukemia screening. Cytometry B Clin Cytom. 2018;94(5):658‐661. 29108126 10.1002/cyto.b.21600

[3] Selimoglu‐Buet D, Wagner‐Ballon O, Saada V, et al. Characteristic repartition of monocyte subsets as a diagnostic signature of chronic myelomonocytic leukemia. Blood. 2015;125(23):3618‐3626. 25852055 10.1182/blood-2015-01-620781 PMC4497970

[4] Tarfi S, Harrivel V, Dumezy F, et al. Multicenter validation of the flow measurement of classical monocyte fraction for chronic myelomonocytic leukemia diagnosis. Blood Cancer Journal. 2018;8(11):114. 30429467 10.1038/s41408-018-0146-8 PMC6235983

[5] Valent P. Oligo‐monocytic CMML and other pre‐CMML states: Clinical impact, prognostication and management. Best Pract Res Clin Haematol. 2020;33(2):101137. 32460976 10.1016/j.beha.2019.101137

[6] Itzykson R, Duchmann M, Lucas N, Solary E. CMML: Clinical and molecular aspects. Int J Hematol. 2017;105(6):711‐719. 28455647 10.1007/s12185-017-2243-z

[7] Solary E, Itzykson R. How I treat chronic myelomonocytic leukemia. Blood. 2017;130(2):126‐136. 28572287 10.1182/blood-2017-04-736421

[8] Vetro C, Chiarenza A, Romano A, et al. Prognostic Assessment and Treatment of Primary Gastric Lymphomas: How Endoscopic Ultrasonography Can Help in Tailoring Patient Management. Clinical Lymphoma Myeloma and Leukemia. 2014;14(3):179‐185. 10.1016/j.clml.2013.10.010 24369919

[9] Vetro C, Romano A, Amico I, et al. Endoscopic features of gastro‐intestinal lymphomas: from diagnosis to follow‐up. World J Gastroenterol. 2014;20(36):12993‐13005. 25278693 10.3748/wjg.v20.i36.12993 PMC4177478

[10] Tisi MC, Ferrero S, Dogliotti I, et al. MYC Rearranged Aggressive B‐Cell Lymphomas: A Report on 100 Patients of the Fondazione Italiana Linfomi (FIL). Hemasphere. 2019;3(6):e305. 31976479 10.1097/HS9.0000000000000305 PMC6924554

[11] Elena C, Gallì A, Such E, et al. Integrating clinical features and genetic lesions in the risk assessment of patients with chronic myelomonocytic leukemia. Blood. 2016;128(10):1408‐1417. 27385790 10.1182/blood-2016-05-714030 PMC5036538

[12] Soriano PK, Stone T, Baqai J, Sana S. A Case of Synchronous Bone Marrow Chronic Myelomonocytic Leukemia (CMML) and Nodal Marginal Zone Lymphoma (NMZL). Am J Case Rep. 2018;19:1135‐1139. 30254190 10.12659/AJCR.910583 PMC6180902

[13] Abdel‐Wahab O, Gao J, Adli M, et al. Deletion of Asxl1 results in myelodysplasia and severe developmental defects in vivo. J Exp Med. 2013;210(12):2641‐2659. 24218140 10.1084/jem.20131141 PMC3832937

[14] Jaiswal S, Fontanillas P, Flannick J, et al. Age‐related clonal hematopoiesis associated with adverse outcomes. N Engl J Med. 2014;371(26):2488‐2498. 25426837 10.1056/NEJMoa1408617 PMC4306669

[15] Genovese G, Kähler AK, Handsaker RE, et al. Clonal hematopoiesis and blood‐cancer risk inferred from blood DNA sequence. N Engl J Med. 2014;371(26):2477‐2487. 25426838 10.1056/NEJMoa1409405 PMC4290021

[16] Barresi V, Romano A, Musso N, et al. Broad copy neutral‐loss of heterozygosity regions and rare recurring copy number abnormalities in normal karyotype‐acute myeloid leukemia genomes. Genes Chromosomes Cancer. 2010;49(11):1014‐1023. 20725993 10.1002/gcc.20810

[17] Barresi V, Palumbo GA, Musso N, et al. Clonal selection of 11q CN‐LOH and CBL gene mutation in a serially studied patient during MDS progression to AML. Leuk Res. 2010;34(11):1539‐1542. 20674974 10.1016/j.leukres.2010.07.004

[18] Palumbo GA, Parrinello NL, Giallongo C, et al. Monocytic Myeloid Derived Suppressor Cells in Hematological Malignancies. Int J Mol Sci. 2019;20(21). 10.3390/ijms20215459 PMC6862591 31683978

